# Neurology: Marine Toxin Hinders Cognitive Development

**DOI:** 10.1289/ehp.114-a94

**Published:** 2006-02

**Authors:** Carol Potera

Domoic acid, a naturally occurring marine toxin, causes acute symptoms of diarrhea, vomiting, seizures, and memory loss in people sickened by eating contaminated shellfish. Now a recent study reveals that exposure to even tiny amounts of domoic acid *in utero* may produce subtle, long-term cognitive impairment in rats. The new findings raise the possibility that pregnant women who inadvertently eat shellfish tainted with low levels of domoic acid may put their unborn children at risk for lifelong behavioral consequences, says Edward D. Levin, a professor of psychiatry at Duke University Medical Center in Durham, North Carolina. Levin coauthored the study, which is described in the September/ October 2005 issue of *Neurotoxicology and Teratology*.

Harmful algal blooms that produce domoic acid are increasing, possibly due to warming ocean waters and human impacts such as farm and sewage runoff. Shellfish take in the toxin when they filter water and incorporate it into their tissues. Along the Florida coast, pigments in the “red tides” caused by *Karenia brevis* and other dinoflagellates signal toxic blooms. However, no distinctive color characterizes toxic blooms elsewhere, such as those along the Oregon coast caused by *Pseudonitzschia*, a phytoplankton that generates domoic acid. “The first warning sign we get for these toxic blooms is when domoic acid shows up in routine testing of shellfish,” says Peter Strutton, a biological oceanographer at Oregon State University in Corvallis. Thus, it’s possible that shellfish with potentially dangerous levels of domoic acid are being harvested and consumed (Strutton notes that, in Oregon at least, most of the shellfish of concern are those that people catch recreationally on their own).

In the Levin study, the researchers injected pregnant rats with single doses of 0.3, 0.6, or 1.2 milligrams of domoic acid per kilogram body weight at the end of the second trimester. The highest dose was in the low end of the range known to cause acute illness in rats.

During adolescence and adulthood, the offspring underwent a battery of behavioral tests. In a radial-arm maze, which looks like a wagon wheel without its rim, the rats searched for sugary cereal at the ends of arms extending from a central hub. Once eaten, the cereal is not replaced, and the rats must remember which arms they’ve already explored. This test therefore measures working memory. The females performed the same regardless of dose, while the males performed progressively worse as the dose increased (male rats normally perform better than females in this maze).

Next Levin gave the rats low doses of scopolamine, a drug that causes amnesia and memory impairment. Slightly stressing the brain with a low dose of scopolamine helped to uncover subtle neurological defects caused by domoic acid. Compared to controls, rats exposed to domoic acid had greater memory loss following administration of scopolamine, with the highest-dose group performing the worst. “Animals can normally deal with a low dose of scopolamine,” says Levin, “unless there’s prior neurotoxic damage that adversely affects the brain.”

Now researchers wonder whether domoic acid may negatively affect unborn children even at levels that do not cause symptoms in expectant mothers. The U.S. Food and Drug Administration based its current limits for domoic acid in shellfish on levels that are assumed to be safe for adults. “We may need to re-evaluate the monitoring of waters and seafood to make sure that the most sensitive members of the population are protected from toxic exposure to domoic acid,” says Levin. However, he adds, it’s important to ensure that fisherman are not unnecessarily cut off from their livelihood and that people are not deprived of the nutritional benefits of uncontaminated seafood.

Strutton and Michelle Wood at the University of Oregon in Eugene are developing a new tool to improve early surveillance of toxic blooms. They are combining satellite data on physical attributes of the ocean such as water color and surface temperature to identify early markers for toxic blooms. In collaboration with the National Oceanic and Atmospheric Administration’s CoastWatch program, they plan to develop products for coastal managers such as charts of conditions that raise the risk of domoic acid poisoning of shellfish. CoastWatch also plans to post satellite maps of regions where blooms exist or are developing on its website. “It will warn [managers] to ramp up their shoreline sampling of shellfish beds,” Strutton says.

## Figures and Tables

**Figure:**
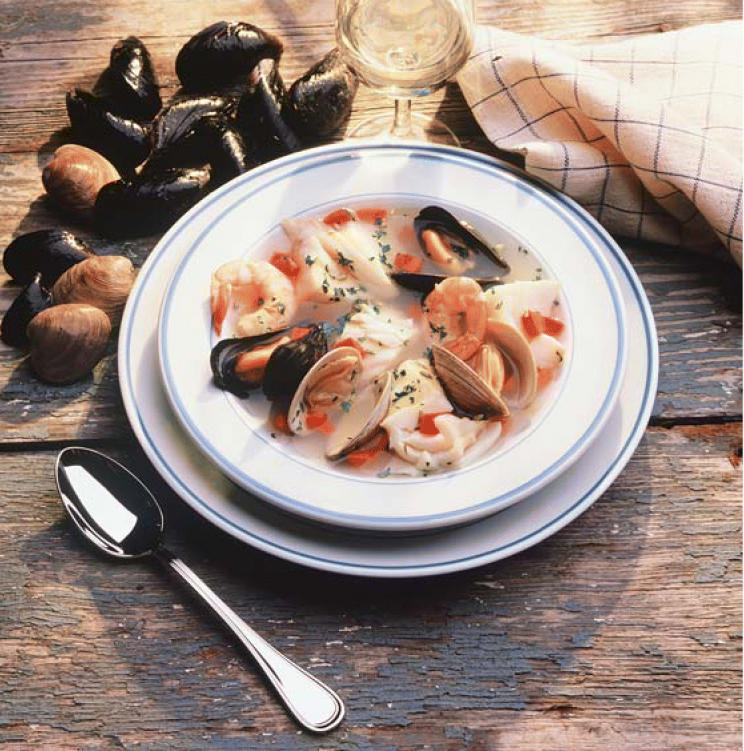
A meal for mothers to skip? New studies in rats show that the marine toxin domoic acid may impair fetal cognitive development when mothers consume contaminated shellfish.

